# Head-to-Head Evaluation of Five Automated SARS-CoV-2 Serology Immunoassays in Various Prevalence Settings

**DOI:** 10.3390/jcm10081605

**Published:** 2021-04-10

**Authors:** Diego O. Andrey, Sabine Yerly, Benjamin Meyer, Isabelle Arm-Vernez, Pascale Roux-Lombard, Giuseppe Togni, Idris Guessous, Hervé Spechbach, Silvia Stringhini, Thomas Agoritsas, Jérôme Stirnemann, Jean-Luc Reny, Claire-Anne Siegrist, Isabella Eckerle, Laurent Kaiser, Nicolas Vuilleumier

**Affiliations:** 1 Division of Laboratory Medicine, Department of Diagnostics, Geneva University Hospitals and Geneva University, 1211 Geneva, Switzerland; Sabine.Yerly@hcuge.ch (S.Y.); Isabelle.Arm-Vernez@hcuge.ch (I.A.-V.); Pascale.Roux-Lombard@hcuge.ch (P.R.-L.); Laurent.kaiser@hcuge.ch (L.K.); nicolas.vuilleumier@hcuge.ch (N.V.); 2 Division of Infectious Diseases, Department of Medicine, Geneva University Hospitals, 1211 Geneva, Switzerland; Isabella.Eckerle@hcuge.ch; 3 Geneva Centre for Emerging Viral Diseases, Geneva University Hospitals, 1211 Geneva, Switzerland; 4 Centre for Vaccinology, Department of Pathology and Immunology, University of Geneva, 1205 Geneva, Switzerland; benjamin.meyer@unige.ch (B.M.); claire-anne.siegrist@unige.ch (C.-A.S.); 5 Division of Immunology, Geneva University Hospitals, 1211 Geneva, Switzerland; 6 Unilabs, Central Laboratory Collection Centers, 1296 Coppet, Switzerland; giuseppe.togni@unilabs.com; 7 Division and Department of Primary Care Medicine, Geneva University Hospitals, 1211 Geneva, Switzerland; idris.guessous@hcuge.ch (I.G.); herve.spechbach@hcuge.ch (H.S.); Silvia.Stringhini@hcuge.ch (S.S.); 8 Unit of Population Epidemiology, Division of Primary Care, Geneva University Hospitals, 1211 Geneva, Switzerland; 9 Division of General Internal Medicine, Department of Medicine, Geneva University Hospitals, 1211 Geneva, Switzerland; Thomas.Agoritsas@hcuge.ch (T.A.); Jerome.Stirnemann@hcuge.ch (J.S.); Jean-luc.reny@hcuge.ch (J.-L.R.); 10 Department of Childhood and Adolescence, Geneva University Hospitals, 1211 Geneva, Switzerland

**Keywords:** SARS-CoV-2, COVID-19, serology, electrochemiluminescent immunoassay (ECLIA), enzyme-linked immunosorbent assay (ELISA), Euroimmun, Roche, Diasorin, Epitope, anti-S, anti-N

## Abstract

Purpose: To assess the diagnostic performances of five automated anti-SARS-CoV-2 immunoassays, Epitope (N), Diasorin (S1/S2), Euroimmun (S1), Roche N (N), and Roche S (S-RBD), and to provide a testing strategy based on pre-test probability. Methods: We assessed the receiver operating characteristic (ROC) areas under the curve (AUC) values, along with the sensitivity, specificity, positive predictive values (PPVs), and negative predictive values (NPVs), of each assay using a validation sample set of 172 COVID-19 sera and 185 negative controls against a validated S1-immunofluorescence as a reference method. The three assays displaying the highest AUCs were selected for further serodetection of 2033 sera of a large population-based cohort. Results: In the validation analysis (pre-test probability: 48.1%), Roche N, Roche S and Euroimmun showed the highest discriminant accuracy (AUCs: 0.99, 0.98, and 0.98) with PPVs and NPVs above 96% and 94%, respectively. In the population-based cohort (pre-test probability: 6.2%) these three assays displayed AUCs above 0.97 and PPVs and NPVs above 90.5% and 99.4%, respectively. A sequential strategy using an anti-S assay as screening test and an anti-N as confirmatory assays resulted in a 96.7% PPV and 99.5% NPV, respectively. Conclusions: Euroimmun and both Roche assays performed equally well in high pre-test probability settings. At a lower prevalence, sequentially combining anti-S and anti-N assays resulted in the optimal trade-off between diagnostic performances and operational considerations.

## 1. Introduction

Determination of the antibody response against SARS-CoV-2 is a common strategy to monitor the prevalence of SARS-CoV-2 exposure populations across the world [1,2,3,4,5,6]. In specific contexts, SARS-CoV-2 serologies might be instrumental for acute diagnostic purposes, particularly when the RT-PCR fails to identify SARS-CoV-2, for example in cases of suboptimal specific pre-analytical situations [7,8]. In certain frequent challenging diagnostic scenarios, such as the presence of pneumonia with an evocative CT-scan but a negative nasopharyngeal SARS-CoV-2 RT-PCR, documentation of a seroconversion with sera obtained days or weeks apart can help confirm the diagnosis [9,10]. Numerous COVID-19 immunoassays have thus been developed to assess anti-SARS-CoV-2 antibody response, and enzyme-linked immunosorbent assay (ELISA)/ electrochemiluminescent immunoassay (ECLIA) tests constitute the current analytical standard to quantify these antibodies. Several CE IVD-marked, fully automated SARS-CoV-2 serological assays are currently available on the market. There are substantial differences among them, not only regarding the kind of antibody isotypes detected (IgG, IgA, IgM or total antibodies), but also in terms of the antigen(s) detected, varying between full trimeric Spike protein (S), its specific S1 or S2 subdomains, the receptor binding domain (RBD) located on S1, the nucleocapsid (N), or a combination thereof [2,11,12,13].

Such differences may offer the opportunity to consider a combined use of some of these assays in a rule-out-then-rule-in strategy to maximize the COVID-19 diagnostic yield. As most European countries are currently facing winter epidemic wave(s) overlapping with the start of massive vaccination programs, the development of appropriate testing strategies will be paramount for the optimization of (i) COVID-19 diagnostic resources allocations and (ii) testing protocols adapted to the swift evolution of pre-test probabilities characterizing the different phases of an epidemic wave.

In the present evaluation, we first performed a head-to-head comparison of the diagnostic accuracy of five immunoassays, including those from Epitope Diagnostics, DiaSorin, Euroimmun, and Roche Diagnostics (both Roche N and Roche S-RBD assays). Next, we evaluated the performance of the three most accurate assays on a large COVID-19 seroprevalence study sampling [1]. We thus sought to define serodiagnostic strategies adapted to either high- or low-prevalence scenarios.

## 2. Methods

### 2.1. Study Population

Negative control serum samples (*n* = 185) were collected for various serological testing in our routine laboratory and stored for analytical validation. These sera were collected in 2018 before the start of the COVID-19 pandemic. Sera (*n* = 172) of PCR-confirmed COVID-19 patients were collected at the University Hospitals of Geneva (HUG), including both hospitalized (*n* = 100) and outpatient clinic (*n* = 72) symptomatic patients. The number of days from symptom onset to blood collection was based on either patient history whenever this information was available, or the date of PCR positivity (*n* = 49).

A second cohort was tested that included 2033 sera from the SEROCoV-POP serosurvey drawn in April 2020. The SEROCoV-POP study is a population-based study from the general population of Geneva, Switzerland. Details regarding the full SEROCoV-POP study are available in the original publication by Stringhini et al. [1].

Ethical approval for sera used in this study was obtained from the local ethics committee of the HUG that approves usage of leftover patient serum collected for diagnostic purposes in accordance with Swiss Regulations on human research. STARD (Standard for Reporting Diagnostic Accuracy Studies) guidelines were followed.

### 2.2. SARS-CoV-2 Analyses

SARS-CoV-2 RT-PCRs were performed as previously published [14,15]. We assessed anti-SARS-CoV-2 antibodies using five commercially available immunoassays according to manufacturer instructions: (1) LIAISON SARS-CoV-2 S1/S2 IgG ELISA on the LIAISON XL analyzer (Diasorin, Vercelli, Italy), (2) EDI Novel Coronavirus COVID-19 IgG ELISA (Epitope Diagnostics, San Diego, CA, USA) on the DSX analyzer (Dynex, Bettlach, Switzerland), (3) Anti-SARS-CoV-2 IgG ELISA (Euroimmun, Lübeck, Germany) on the Agility analyzer (Dynex, Bettlach, Switzerland), (4) Elecsys Anti-SARS-CoV-2 N (anti-N total antibodies) on the Cobas e801 analyzer (Roche Diagnostics, Switzerland) and (5) Elecsys Anti-SARS-CoV-2 S (anti-S1-RBD total antibodies) on the Cobas e801 analyzer, hereafter referred to as Epitope, Diasorin, Euroimmun, Roche N and Roche S. Results are reported as numeric values in the form of an index (signal sample/signal calibrator), interpreted as qualitative results according to the manufacturers’ cut-off for Epitope, Diasorin, Euroimmun and Roche N, and as concentration (U/mL) for the quantitative Roche S assay (Table 1).

As reference method, we used an in-house recombinant immunofluorescence assay (rIFA) to detect IgG antibody response against the complete spike (S) protein (both S1 and S2 domains) of SARS-CoV-2, as previously validated for MERS-CoV [16,17] and adapted to SARS-CoV-2 [18]. The rIFA results were assessed by two independent readers, blinded to the COVID-19 status, with a good inter-observer kappa coefficient [15,18].

### 2.3. Statistical Analyses

We evaluated the overall test performances by conducting receiver operating characteristic (ROC) curve analyses according to the DeLong non parametric test [19] using Analyse-it software for Excel (Analyse-it Software, Ltd., Leeds, UK). We calculated sensitivity, specificity, conventional (not prevalence-weighted) likelihood ratios (LRs), positive predictive values (PPVs) and negative predictive values (NPVs), either against the COVID-19 status (positive RT-PCR) or against a positive rIFA as a reference method, for each serological assay by using MedCalc software 19.2.1 (MedCalc Ltd., Ostend, Belgium). Performance was calculated using manufacturers’ cut-offs and borderline (grey zone) results (for Epitope, Diasorin and Euroimmun) were considered as negative for both the sensitivity and specificity analyses.

## 3. Results

### 3.1. Overall Diagnostic Performance of Five ELISA/ECLIA Performances on COVID-19 Positive and Negative Sera

We used a combined panel of 357 sera, 185 from SARS-CoV-2 naive individuals drawn in 2018 and 172 from patients (39% female, median age: 52 years; range: 14–96) with a positive SARS CoV-2 PCR result from a respiratory specimen. These sera were collected in a median 19 days (range 3 to 39) after the onset of symptoms. The COVID-19 pre-test probability of this combined cohort, by design, was 48%. Additional characteristics of these cohorts are reported in Table 2.

To obtain an evaluation of the overall diagnostic performance of these tests (free of manufacturers’ cut-off influence), we initially performed ROC analyses. As shown in Table 3, Roche N total Ig, Euroimmun IgG, and Roche S total Ig assays were the most accurate in discriminating between COVID-19 cases and controls, with ROC areas under the curve (AUC) of 0.993, 0.982, and 0.977, respectively. Epitope showed an AUC of 0.970 and Diasorin 0.929. AUC comparisons according to the Delong method indicated that the Roche N assays were superior to every other tested immunoassay, and Roche S, Euroimmun, and Epitope performed better than Diasorin (*p* < 0.05) (Supplementary Table S1). Within the same case-control cohorts, when compared to the in-house rIFA as the gold standard, Euroimmun, Roche S and Roche N assays also displayed the highest numerical diagnostic accuracies among the five assays tested, with respective AUCs of 0.996, 0.996, and 0.997, and were not found to differ significantly, according to the Delong method (Table 4, and Supplementary Table S2). These AUCs were, however, superior over those of Diasorin and Epitope (for Roche N and Euroimmun).

We then assessed their sensitivity, specificity, positive and negative likelihood ratios, NPVs, and PPVs with the manufacturers’ cut-off against the in-house rIFA (Table 5). The Roche S showed the highest sensitivity (96.3%) while Roche N and Euroimmun showed sensitivity above 90% with Diasorin and Epitope performing lower. Roche N showed the highest specificity at 99% but all assays showed specificity above 96.9%. Importantly, the NPVs of both Roche assays and the Euroimmun assay were above 94%, and the LRs were below the 0.1 nominal value with respective values of 0.04 and 0.08. The performances of these automated assays when using COVID-19 cases and controls as references (in contrast to positive rIFA results) are shown in Supplementary Table S3. As previously reported [18], the in-house rIFA showed a level of sensitivity (93.6%) and specificity (100%) above any of the automated assays we evaluated in a setting with a median time from symptoms onset to blood collection of 19 days but as low at 3 days.

**Figure 1:**
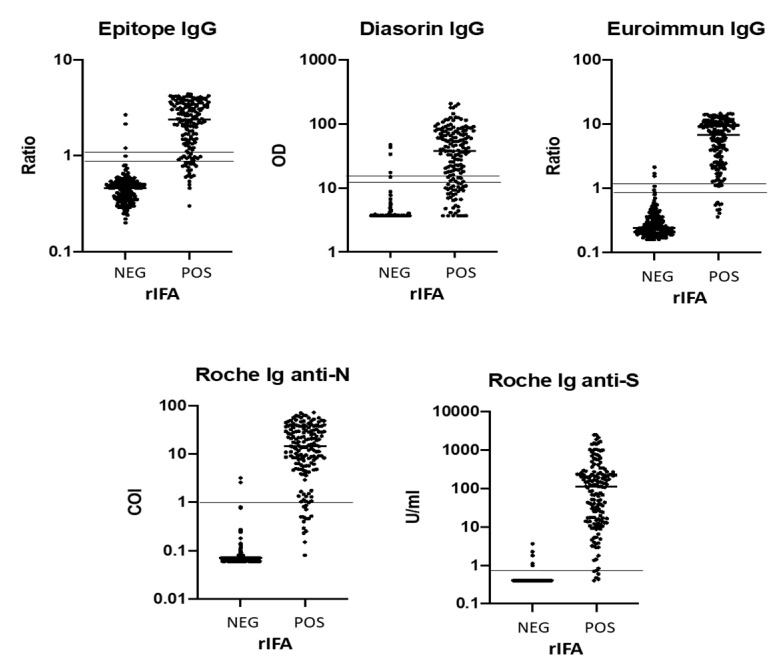
ELISA Ig data distribution according to negative and positive rIFA results. rIFA, recombinant immunofluorescence assay; COI, cut-off index.

The distribution of positive and negative data points showed better separation for Euroimmun and Roche assays. The largest dynamic range was observed for the quantitative Roche S assay (Figure 1).

### 3.2. Comparison of Euroimmun and both Roche Assays Performance in a General Population-Based Cohort

Due to these results and to the close to optimal AUCs of Euroimmun, Roche N, and Roche S assays, these three assays were selected for validation and for a further comparative analysis using sera collected in the context of a recent general population-based seroprevalence survey. Sera from 2033 individuals, collected over 4 weeks in April 2020, were included in this study. All sera found positive either with Roche N, Roche S, or found positive or borderline (cut-off IgG ratio 0.8–1.1) by Euroimmun IgG assays were subsequently tested with rIFAs, as a reference method. Sera found negative by all three assays, were not confirmed with rIFAs due to the high NPVs and negative LRs shown for these assays in the high pre-test probability cohort.

The prevalence of anti-SARS-CoV-2 seropositivity based on rIFA testing was 6.2% (*n* = 126, 95% CI, 5.19–7.34) and ROC analyses yielded AUCs of 0.988, 0.983, and 0.967 for Euroimmun, Roche, N, and Roche S, respectively. While 127 (6.25%) sera were classified as IgG positive with the Euroimmun assay (cut-off > or = 1.1), antibodies were detected in 122 (6.0%) and 136 (6.66%) with the Roche N and S assays, respectively. Euroimmun, Roche N, and Roche S sensitivity was 91.3%, 92.9%, and 100%, respectively (Table 6). Specificity was above 99% for the two Roche assays and Euroimmun (Table 6).

In this study of a population with low disease prevalence, PPVs ranged from 90.55% for Euroimmun to 92.7% and 95.9% for Roche S and Roche N, respectively. NPVs reached 100% for the Roche S assay and more than 99% for the Euroimmun and Roche N assays.

### 3.3. Assessment of Different Testing Strategies on the Population-Based Cohort

Different strategies were analyzed in order to provide accurate antibody results without the use of time-consuming rIFA confirmatory tests in the population cohort. We assessed a sequential strategy using an initial screening assay followed by a confirmatory assay for positive samples. We included every possible combination with the Roche N, Roche S and Euroimmun assays. We also tested the performances of a strategy using the parallel detection of antibodies against both antigens (Roche N and Roche S). Both cut-offs provided by Euroimmun were used to either maximize sensitivity (as a screening assay) or specificity (as a confirmatory assay). The results are shown in Table 6. Our analysis showed that the PPV could be maximized to 100% using either Roche S or Roche N as a screening assay followed by Euroimmun (cut-off 1.1 to maximize specificity) as a confirmatory assay while maintaining an NPV above 99%. Sequential use of Roche S followed by Roche N confirmation provided a PPV of 96.3% and an NPV of 99.5%, values within the 95% CI of the previous option. In comparison, either Roche S alone or Roche S and Roche N parallel testing (either one positive leading to a positive result) displayed an NPV of 100%. 

## 4. Discussion

Among the five automated serological assays tested here, those from Euroimmun and Roche outperformed Diasorin, regardless of the gold standard considered (rIFA or RT-PCR-based diagnosis) in high prevalence scenarios, while the Roche N assay also outperformed Epitope, based on AUC comparisons. Furthermore, at the manufacturer cut-offs, Diasorin and Epitope assays displayed suboptimal NPVs for confident rule-out in high pre-test probability situations, as the low end of the 95% CI observed was 77.9%. These results are in line with a recently published study indicating that the Roche N assay encompassed the highest AUC among six existing solutions [20,21]. Taken together, these results indicate that in the specific situation of high prevalence, for any of the Euroimmun or Roche assays, their NPVs and PPVs were found to be sufficiently high (above 94%) to consider their use as a single test both for rule-in and rule-out purposes without requiring the need of a confirmatory assay. These results are an extension of previous reports performed in variable pre-test probabilities settings [13,20,21,22,23,24,25,26,27].

Furthermore, although Roche S ECLIA has been used in a major seroprevalence study in Switzerland, this present study is the first to test its diagnostic performance in both low and high pre-test probability settings [28]. Our results indicate that S-assays tend to display slightly better sensitivity and NPV. The higher sensitivity of the Roche S compared to Roche N held true even in the subgroup of sera (*n* = 50) drawn earlier after symptoms onset (range 3 to 14 days). This contrasts with published results suggesting that anti-N assays showed better accuracy to detect early seropositivity [20,29].

Building upon these initial results, we tested these three assays in a general population setting consisting in 2033 individuals with a COVID-19 prevalence of 6.2%. As expected, the respective PPV of these assays using the manufacturers cut-offs substantially decreased, despite displaying high AUCs, while the NPVs were above 99.5% with the lowest end of the 95% CI at 99.0% observed for the Euroimmun assay. In this context, all possible assay combinations were considered. Among these, the combination of any of Roche assay for screening purposes with Euroimmun as a confirmatory assay was found to provide the optimal diagnostic performance with an optimal PPV of 100% and an NPV above 99%. This sequence would meet the CDC’s recommendation of targeting a PPV greater than 99.5%. An additional noteworthy benefit of combining serology platforms using both anti-N and anti-S antibody detection systems would be the detection antibodies against diverse antigens and thus differentiate antibodies induced by SARS-CoV-2 infection (with both anti-S and anti-N antibodies) versus vaccine-induced antibodies (only anti-S antibodies). S-based vaccine strategies would display an anti-RBD positive combined with an anti-N negative profile. It is important to note that these combined solutions can easily be adapted to the evolving prevalence, whether due to the successive epidemic waves or to a vaccine-induced immunity. Importantly, with the progression of the epidemic and vaccines being rolled out, high-prevalence scenarios will become likely.

Limitations: although we performed the rIFA confirmatory assay on every borderline or positive result of the 2033 population sera to avoid false-positives, rIFA could not be carried out in all negative sera due to the heavy workload. We believe, nevertheless, that the bias is minimal, since we showed that these assays have a high NPV (around 95%) in the high pre-test probability validation dataset. They also displayed an excellent PPV even at the high pre-test probability of the validation dataset. False-negatives are thus probably negligible in the low-prevalence population cohort. We also must stress that this study was performed with sera of individuals infected early during the pandemic, before the circulation of SARS-CoV-2 variants. Whether the performance of these serological assays is altered after infection with a SARS-CoV-2 mutant viral strain remains uncertain (compared to the initial strain) and this study cannot clarify this potential issue. To the best of our knowledge, we found no published data suggesting a sensitivity detection issue (increased false-negatives), but this remains to be formally addressed, especially with RBD-based capture antigen assays. Finally, another limitation is that we did not evaluate several other automated systems that currently exist, although some have reported varying performances, such as Oxford immunoassays, Siemens or Abbott [21].

In conclusion, among these five automated antibody tests, Euroimmun and both Roche assays performed equally well in high and low pre-test probability settings. In high-prevalence settings, either Roche S or Euroimmun assays could be considered without a secondary confirmatory test for both rule-in and rule-out purposes. In lower pre-test probability settings, sequentially combining anti-S followed by anti-N assays resulted in the optimal trade-off between diagnostic performance and operational considerations.

## Figures and Tables

**Table 1:** Serological assays used in the study.

Assay	Manufacturer	Method	Antibody	Antigen	Cut-off
EDI Novel Coronavirus COVID-19	Epitope Diagnostic	ELISA	IgG	N	0.9–1.1
LIAISON SARS-CoV-2	Diasorin	ELISA	IgG	S1/S2	12–15
SARS-CoV-2	Euroimmun	ELISA	IgG	S1	0.8–1.1
Elecsys Anti-SARS-CoV-2 N	Roche	ECLIA	Ig total	N	≥1.0
Elecsys Anti-SARS-CoV-2 S	Roche	ECLIA	Ig total	S1-RBD	≥0.80
Recombinant Immunofluorescence	In-house	rIFA	IgG	S1/S2	Neg/Pos

rIFA: recombinant immunofluorescence assay. ELISA: enzyme-linked immunosorbent assay; ECLIA: electrochemiluminescent immunoassay.

**Table 2:** Characteristics of the cohorts.

Cohorts	*N*	Date	COVID-19
Pre-epidemic individuals	185	2018	Naive
Hospitalized/outpatients	172	March–April 2020	RT-PCR confirmed
General population	2033	April 2020	Unknown

**Table 3:** Receiver operating characteristic (ROC) curves of the five tested commercial assays (COVID-19 cases versus controls).

Assays	AUC	95% CI	*p* Value
Epitope	0.970	0.950–0.990	<0.0001
Diasorin	0.929	0.896–962	<0.0001
Roche N	0.993	0.985–1.00	<0.0001
Roche S	0.977	0.961–0.993	<0.0001
Euroimmun	0.982	0.969–0.994	<0.0001

AUC: Area Under the Curve.

**Table 4:** ROC curves of the five tested commercial assays against rIFA.

Assays	AUC	95% CI	*p* Value
Epitope	0.980	0.965–0.995	<0.0001
Diasorin	0.964	0.941–0.987	<0.0001
Roche N	0.997	0.994–1.000	<0.0001
Roche S	0.996	0.990–1.000	<0.0001
Euroimmun	0.996	0.992–0.999	<0.0001

**Table 5:** Sensitivity, specificity, PPV and NPV of tested assays (vs. rIFA).

	Epitope *		Diasorin *		Roche N		Roche S		Euroimmun *	
	Value	95% CI	Value	95% CI	Value	95% CI	Value	95% CI	Value	95% CI
Sensitivity	82.00%	75.2–87.6	73.90%	66.4–80.5	92.60%	87.3–96.1	96.30%	92.1–98.7	92.60%	87.3–96.1
Specificity	98.50%	95.6–99.7	98.50%	95.6–99.7	99.00%	96.4–99.9	97.50%	94.2–99.2	98.47%	95.59–99.68
PLR	53.6	17.4–165.0	48.3	15.7–149.0	90.7	22.83–360.34	37.74	15.88–89.70	60.46	19.65–186.01
NLR	0.18	0.13–0.25	0.26	0.20–0.34	0.08	0.04–0.13	0.04	0.02–0.08	0.08	0.04–0.13
PPV	97.78%	93.46–99.27	97.54%	92.78–99.19	98.68%	94.94–99.66	96.88%	92.88–98.66	98.03%	94.17–99.35
NPV	86.94%	82.71–90.25	82.13%	77.98–85.64	94.17%	90.37–96.54	96.95%	93.55–98.59	94.15%	90.32–96.52

* Borderline samples were handled as negative. PLR, positive likelihood ratio; NLR, negative likelihood ratio; PPV, positive predictive value; NPV, negative predictive value; rIFA, recombinant Immunofluorescence assay.

**Table 6:** Sensitivity, specificity, PPV and NPV of different testing strategies in the seroprevalence study.

	Roche N Alone		Roche S Alone		Euroimmun Alone		Sequential Roche S/confirmatory Roche N		Parallel Roche S and Roche N		Sequential Roche S/confirmatory EI (≥1.1)		Sequential Roche N/confirmatory EI (≥1.1)		Sequential EI (≥0.8)/confirmatory Roche S		Sequential EI (≥0.8)/confirmatory Roche N	
Statistic	Value	95% CI	Value	95% CI	Value	95% CI	Value	95% CI	Value	95% CI	Value	95% CI	Value	95% CI	Value	95% CI	Value	95% CI
Sensitivity	92.86%	86.87–96.68	100.00%	97.11–100.00	91.27%	84.56–95.35	92.86%	86.87–96.68	100.00%	97.11–100.00	91.27%	84.92–95.56	92.86%	86.87–96.68	96.03%	90.98–98.70	91.27%	84.92–95.56
Specificity	99.74%	99.39–99.91	99.48%	99.04–99.75	99.37%	98.90–99.67	99.79%	99.46–99.94	99.42%	98.97–99.71	100.00%	99.81–100.00	100.00%	99.81–100.00	99.90%	99.62–99.99	99.69%	99.32–99.88
PPV	95.90%	90.69–98.25	92.65%	87.16–95.90	90.55%	83.74–94.81	96.69%	91.65–98.73	91.97%	86.40–95.38	100.00%	99.81–100.00	100.00%	99.81–100.00	98.37%	93.80–99.59	95.04%	89.59–97.71
NPV	99.53%	99.12–99.75	100.00%	99.75–100.00	99.42%	98.95–99.70	99.53%	99.12–99.75	100.00%	99.75–100.00	99.43%	99.00–99.67	99.53%	99.12–99.75	99.74%	99.38–99.89	99.42%	98.99–99.67

PPV, positive predictive value; NPV, negative predictive value; EI, Euroimmun.

## Supplementary Materials

The following are available online at https://www.mdpi.com/article/10.3390/jcm10081605/s1, Table S1: ROC curves statistics (vs. COVIS-19 cases/controls); Table S2: ROC curves statistics (vs. rIFA). Table S3: Sensitivity, specificity, PPV, NPV and accuracy of tested assays (COVID-19 cases versus controls).
