# Supplementary material for: Head-to-Head Evaluation of Five Automated SARS-CoV-2 Serology Immunoassays in Various Prevalence Settings
Source: J Clin Med. 2021 Apr 10;10(8):1605. doi: 10.3390/jcm10081605 (PMC8069412; doi:10.3390/jcm10081605)
Supplement: Supplementary file 1 [file jcm-10-01605-s001.pdf]

Supplementary Table S1. ROC curves statistics (vs. COVID-19 cases/controls).

Assays	Difference	95% CI		SE	Z	*p*-Value
Roche N–DiaSorin	0.063	0.033	to 0.094	0.0155	4.09	<0.0001
EI–DiaSorin	0.052	0.022	to 0.082	0.0153	3.42	0.0006
Roche S1–DiaSorin	0.047	0.020	to 0.075	0.0138	3.43	0.0006
Epitope–DiaSorin	0.040	0.009	to 0.072	0.0162	2.50	0.0123
Roche N–Epitope	0.023	0.007	to 0.039	0.0081	2.83	0.0046
Roche N–Roche S1	0.016	0.003	to 0.029	0.0065	2.44	0.0146
EI–Epitope	0.012	−0.007	to 0.031	0.0097	1.22	0.2227
Roche N–EI	0.011	0.001	to 0.021	0.0051	2.15	0.0319
Roche S1–Epitope	0.007	−0.012	to 0.026	0.0098	0.71	0.4747
EI–Roche S1	0.005	−0.006	to 0.015	0.0053	0.91	0.3635

Supplementary Table S2. ROC curves statistics (vs. rIFA).

Assays	Difference	95% CI		SE	Z	*p*-Value
Roche N–DiaSorin	0.033	0.010	to 0.056	0.0117	2.83	0.0046
Roche S1–DiaSorin	0.032	0.011	to 0.053	0.0108	2.96	0.0031
EI–DiaSorin	0.032	0.010	to 0.054	0.0112	2.83	0.0047
Roche N–Epitope	0.017	0.003	to 0.030	0.0068	2.46	0.0140
Epitope–DiaSorin	0.016	−0.010	to 0.043	0.0135	1.22	0.2240
Roche S1–Epitope	0.015	−0.001	to 0.031	0.0082	1.90	0.0578
EI–Epitope	0.015	0.000	to 0.030	0.0076	2.01	0.0446
Roche N–EI	0.001	−0.003	to 0.005	0.0020	0.70	0.4821
Roche N–Roche S1	0.001	−0.005	to 0.008	0.0033	0.39	0.6979
Roche S1–EI	0.000	−0.007	to 0.007	0.0035	0.04	0.9713

Supplementary Table S3. Sensitivity, specificity, PPV, NPV and accuracy of tested assays (COVID-19 cases versus controls).

	**Epitope ***		**Diasorin ***		**Roche N**		**Roche S1**		**Euroimmun ***		**rIFA**	
	**Value**	**95% CI**	**Value**	**95% CI**	**Value**	**95% CI**	**Value**	**95% CI**	**Value**	**95% CI**	**Value**	**95% CI**
Sensitivity	77.91%	70.96–83.87	69.19 %	61.71–75.99	87.79%	81.94–92.28	92.44 %	87.42–95.91	89.53 %	83.97–93.68	93.60%	88.85–96.76
Specificity	99.46%	97.03–99.99	97.84 %	94.56–99.41	100.00 %	98.03–100.00	99.46 %	97.03–99.99	98.38 %	94.95–99.58	100.00 %	98.03–100.00
PLR	144.13	20.38–1019.45	32.00	12.08–84.79		NA	171.02	24.21–1208.25	55.21	17.95–169.82		NA
NLR	0.22	0.17–0.29	0.31	0.25–0.39	0.12	0.08–0.18	0.08	0.05–0.13	0.11	0.07–0.17	0.06	0.04–0.11
PPV	99.26%	94.99–99.89	96.75 %	91.82–98.75	100.00 %	96.91–100.00	99.38 %	95.75–99.91	98.09 %	94.08–99.51	100.00 %	97.10–100.00
NPV	82.88%	78.52–86.51	77.35 %	73.17–81.05	89.81%	85.51–92.93	93.40 %	89.35–95.98	91.13 %	86.91–94.09	94.39%	90.47–96.75

* Borderline samples were handled as negative. PLR, positive likelihood ratio; NLR, negative likelihood ratio; PPV, positive predictive value; NPV, negative predictive value
